# Supplementary material for: Tead1a Initiates Transcriptional Priming Through the TEAD1a/YAP‐Notch1‐Spi1/Cebpα Axis to Promote Neutrophil Fate
Source: Adv Sci (Weinh). 2025 Aug 28;12(41):e05441. doi: 10.1002/advs.202505441 (PMC12591127; doi:10.1002/advs.202505441)
Supplement: Supplementary file 4 — Supplementary Dataset [file ADVS-12-e05441-s004.zip › 3-5.zip!/3-5/02-5.cluster/cluster13/Enrich/ego_CC.pdf]

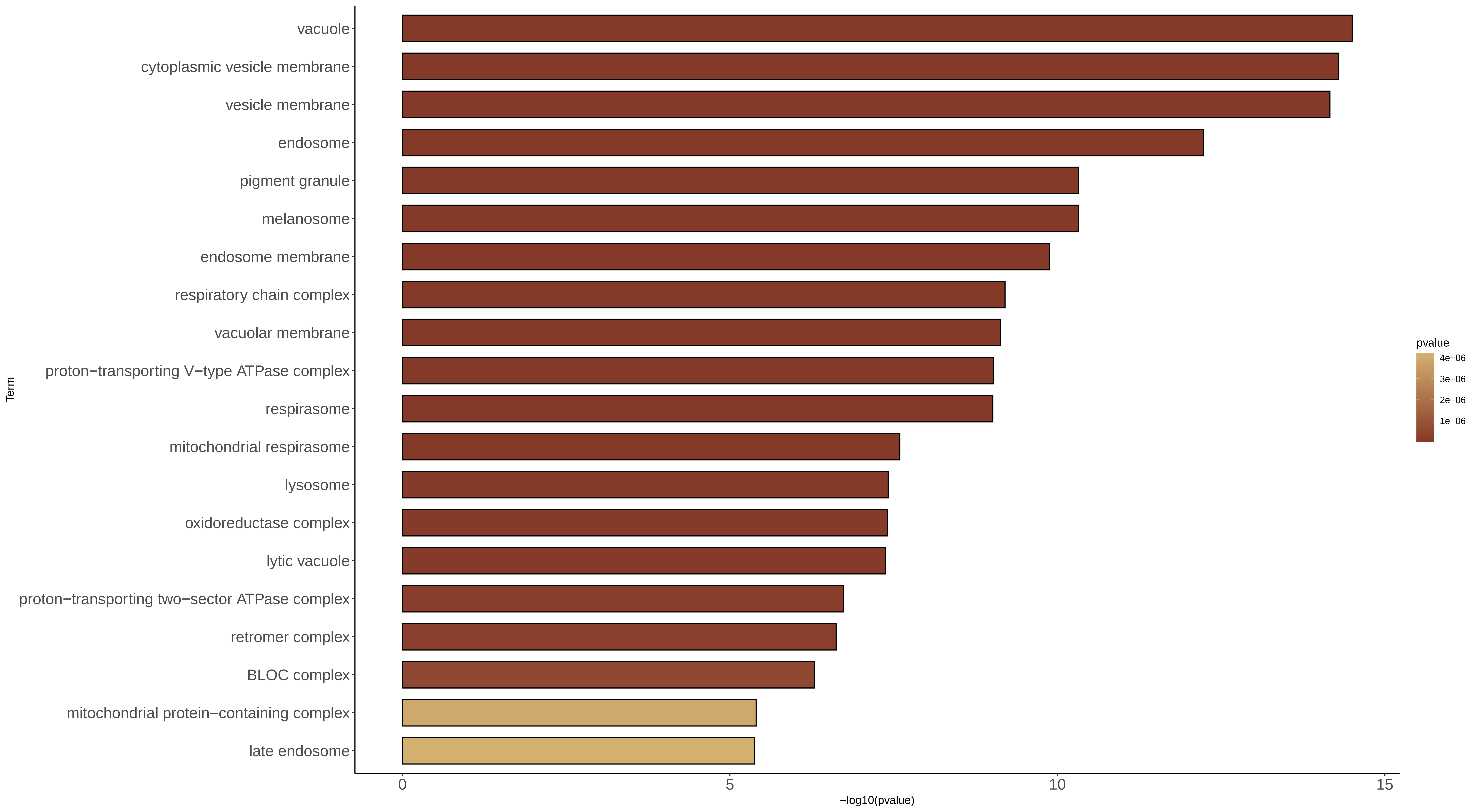

vacuole
cytoplasmic vesicle membrane
vesicle membrane
endosome
pigment granule
melanosome
endosome membrane
respiratory chain complex
vacuolar membrane
proton−transporting V−type ATPase complex
respirasome
mitochondrial respirasome
lysosome
oxidoreductase complex
lytic vacuole
proton−transporting two−sector ATPase complex
retromer complex
BLOC complex
mitochondrial protein−containing complex
late endosome
Term
0
5
10
15
−log10(pvalue)
pvalue
4e−06
3e−06
2e−06
1e−06
